# Availability of Serum Levels of Carbohydrate Antigen 19-9 (CA19-9) as a Surrogate Tumor Marker for Papillary Thyroid Carcinoma

**DOI:** 10.31662/jmaj.2023-0180

**Published:** 2024-02-09

**Authors:** Minoru Kihara, Akira Miyauchi, Mitsuyoshi Hirokawa, Ayana Suzuki, Takashi Akamizu

**Affiliations:** 1 Departments of Surgery, Kuma Hospital, Kobe, Japan; 2 Diagnostic Pathology, Kuma Hospital, Kobe, Japan; 3 Internal Medicine, Kuma Hospital, Kobe, Japan

**Keywords:** papillary thyroid carcinoma, carbohydrate antigen 19-9 (CA19-9), thyroglobulin, tumor marker, thyroglobulin antibody

## Abstract

**Introduction:**

Thyroglobulin (Tg) is a very sensitive and specific marker in patients who have undergone total thyroidectomy for papillary thyroid carcinoma (PTC). However, the presence of a Tg antibody (TgAb) interferes with Tg immunometric assays, making Tg levels unreliable indicators. There are currently no other tumor markers to monitor in patients with PTC whose serum is TgAb-positive. Thus, we investigated whether carbohydrate antigen 19-9 (CA19-9) can be used as a tumor marker for PTC.

**Methods:**

We retrospectively analyzed 196 consecutive patients with PTC (maximum diameter ≥ 2 cm). The serum CA19-9 and Tg values of each patient were obtained before and 0.5-1 month postsurgery. Immunohistochemical staining for PTC was performed using an antibody against CA19-9.

**Results:**

High pre-surgery serum levels of CA19-9 were observed in 6.1% of the patients. Postsurgery, serum CA19-9 levels in all 196 patients decreased considerably and were within the normal range. CA19-9 expression was detected in 28 of 62 PTCs (45.2%) and was detected at various degrees and ranges in conventional PTC histology.

**Conclusions:**

Although further studies with longer follow-ups are necessary, serum CA19-9 levels may serve as a surrogate tumor marker for PTC in place of serum Tg levels sin some patients.

## Introduction

Thyroglobulin (Tg), a glycoprotein with a molecular weight of approximately 660 kDa, is synthesized by thyrocytes and released into the lumen of thyroid follicles ^(1)^. Serum Tg levels are an effective and useful tumor marker for papillary thyroid carcinoma (PTC), particularly in patients who have undergone total thyroidectomy ^(2), (3), (4), (5)^. Postoperative serum Tg value is an important prognostic factor that is used to guide clinical managementand is the most valuable tool in the long-term follow-up of patients with PTC ^(2), (3), (4), (5)^. However, the distribution of Tg values is affected by the presence of Tg antibodies (TgAbs) ^(6), (7), (8), (9), (10), (11)^.

The most commonly used and best-validated serum tumor marker for diagnosing pancreatic cancer in symptomatic patients and for monitoring therapy in patients with pancreatic adenocarcinoma is carbohydrate antigen 19-9 (CA19-9). Elevated CA19‐9 levels have also been observed in gastrointestinal, biliary tract, and ovarian tumors, particularly in the mucinous type. We recently reported the case of a 69-year-old man with multiple metastases of PTC and high serum levels of CA19-9, which indicated the possibility that CA19-9 may be a tumor marker for PTC ^(12)^. However, there are only a few case reports of high serum CA19-9 levels in patients with PTC ^(13), (14), (15), (16)^, and it has not been established whether serum CA19-9 level is a useful tumor marker for PTC.

We conducted this study to determine CA19-9 positivity in PTC tissues and investigate whether CA19-9 can be used as a tumor marker for PTC.

## Materials and Methods

We retrospectively analyzed 196 consecutive patients with PTC (maximum diameter ≥ 2 cm) who underwent total thyroidectomy at Kuma Hospital, Japan from April to November 2018. The patients were 48 males and 148 females aged 16-89 years (median 60 years). Of the 196 patients, 128 (65.3%) underwent total thyroidectomy and central lymph node dissection, and the other 68 (34.7%) underwent total thyroidectomy and both central and lateral lymph node dissections. None of the patients had lung metastases at the time of surgery, and none had diabetes mellitus, hepatic cirrhosis, renal failure, or viral hepatitis.

For each patient, serum CA19-9 levels were measured before and at 0.5-1 month after surgery. Serum Tg and TgAb measurements were performed before, at 0.5-1 month, and at 3 months after surgery. Furthermore, the patients were followed up with serum Tg and TgAb measurements every 6 months and an ultrasound examination every year thereafter. As previously described ^(17)^, we followed up with the patients by conducting imaging studies such as chest roentgenography or computed tomography as well as other imaging studies, if indicated. Serum CA19-9 levels were measured in a laboratory (SRL Co., Tokyo) using the Elecsys^Ⓡ^ CA19-9 II test system (Roche Diagnostics, Tokyo), which is an electrochemiluminescence immunoassay (ECLIA). According to the manufacturer, the normal range of serum CA19-9 is ≤37 U/mL. Immunoassays for Tg and TgAb were performed using the ECLIA method, Elecsys^Ⓡ^ Tg II system, and Elecsys^Ⓡ^ TgAb (Roche Diagnostics). The lower limit of quantification for Tg was 0.08 ng/mL, and the cut-off value for TgAb was 40 IU/mL.

Immunohistochemical staining for PTC was performed using the automated Leica Bond-Max system and Bond Refine detection kit (Leica Microsystems, Wetzlar, Germany) according to the manufacturer’s recommendations fordilution ions. We used a primary antibody against CA19-9 (Clone 1116-NS-19-9, 1:200, No antigen retrieval; Dako, Carpinteria, CA, USA). We defined positive CA19-9 immunostaining as cases with >1% carcinoma cells expressing moderate or strong positive staining. An anti-Ki-67 antibody (MIB-1, 1:200; Dako) was used for the immunocytochemistry.

This study was approved by our hospital’s institutional ethics committee (no. 20180517-1). The patients’ written informed consent for the use of their clinical information for this research was obtained before surgery.

### Statistical analysis

Fisher’s exact probability test, the Wilcoxon signed-rank test, and Mann-Whitney U-test were used for the statistical analyses. Pearson’s correlation coefficient (r) was used to analyze the correlation between the two variables. A *p*-value of <0.05 was considered significant. All analyses were performed using StatFlex 7.0 software (Artech, Osaka, Japan).

## Results

### Serum levels of CA19-9 before and after surgery

**Figure 1.**
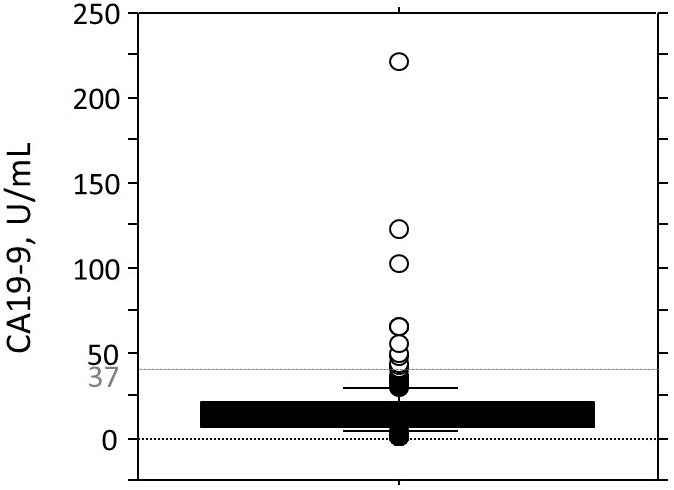
Distribution of serum CA-19-9 levels before the patients’ surgeries. Twelve (6.1%) of the 196 patients with PTC showed high serum CA19-9 levels before surgery. White circles, high serum CA19-9 levels ; Black circles, normal serum CA19-9 levels.

Before the surgeries, 12 (6.1%) of the 196 patients with PTC showed high serum CA19-9 levels (High group; median 60.5 U/mL, range 40.8-221 U/mL), and the remaining 184 patients showed normal serum CA19-9 levels (Normal group; median 11.2 U/mL, range 1.0-36.3 U/mL) (Figure 1). Table 1 shows the summary of the characteristics of the two patient groups. There were no significant differences between the Normal and High groups other than the percentage of cases with lymphangial invasion; the Normal group had a significantly higher number of cases that were negative for lymphangial invasion.

**Table 1.** Clinicopathological Characteristics of Patients with PTC with Normal and High Serum CA19-9 Levels before Surgery.

	Normal serum CA19-9	High serum CA19-9	p-value
No. of patients	184 (93.9%)	12 (6.1%)	
Age, years ^a^	47 (16-89)	38.5 (18-87)	0.531
Gender:			
Male	45 (24.5%)	3 (25.0%)	0.975
Female	139 (75.5%)	9 (75.0%)
Max. tumor size, mm ^a^	26 (20-110)	33 (20-61)	0.130
Anti-thyroglobulin antibody:			
Positive	32	1	0.695
Negative	152	11	
Serum thyroglobulin levels before surgery, ng/mL^ a,b^	59.8 (2.0-2684)	122.0 (1.0-1875)	0.400
Serum thyroglobulin levels after surgery, ng/mL^ a,b^	1.2 (0.1-185)	2.3 (0.3-377.7)	0.308
Extrathyroidal invasion:			
Positive	10	2	0.287
Negative	174	10	
Nodal metastasis:			
Positive	159	11	0.249
Negative	25	1	
Vascular invasion:			
Positive	10	1	0.512
Negative	174	11
Lymphangial invasion:
Positive	4	2	0.046
Negative	180	10	
Ki-67 labeling index:			
0%-5%	119	8	
5%-10%	37	2	0.567
>10%	5	1	
Unknown	23	1
Serum level of CA19-9, U/mL: ^a^			
Before surgery	11.2 (1-36.3)	60.5 (40.8-221)	<0.01
After surgery	9.0 (1-29.5)	32.5 (17.5-36)	<0.01

^a^ Median (range). ^b^ Patients positive for the thyroglobulin antibody were excluded.

**Figure 2.**
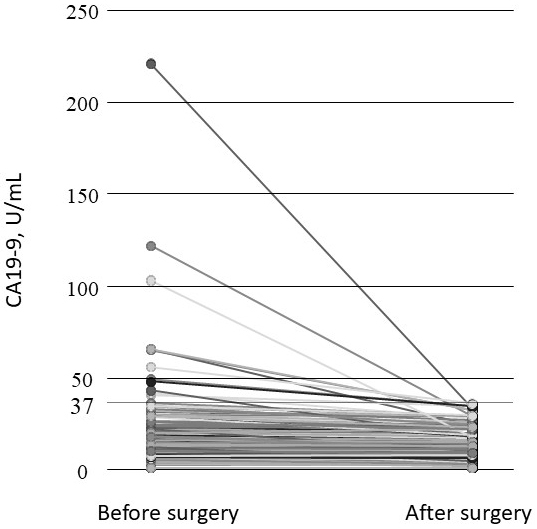
The patients’ serum CA19-9 levels before and after surgery.

Each patient’s serum CA19-9 levels before and after surgery are depicted in Figure 2. The serum CA19-9 levels of all patients in the Normal group remained in the normal range postoperatively, and those of the High group decreased considerably and were within the normal range postoperatively. Thus, no patients in the High or Normal group had a high serum CA19-9 level postoperatively.

There was no significant correlation between serum CA19-9 and Tg levels before and after surgery in patients who are negative for TgAb, based on the Pearson’s correlation coefficient (*r* = 0.12, *p* = 0.18 and *r* = −0.06, *p* = 0.86). In addition, there was no significant correlation between serum CA19-9 and TgAb levels before and after surgery (*r* = 0.49, *p* = 0.75 and *r* = 0.20, *p* = 0.53).

### Serum CA19-9 levels before surgery and immunohistochemical stains

**Figure 3.**
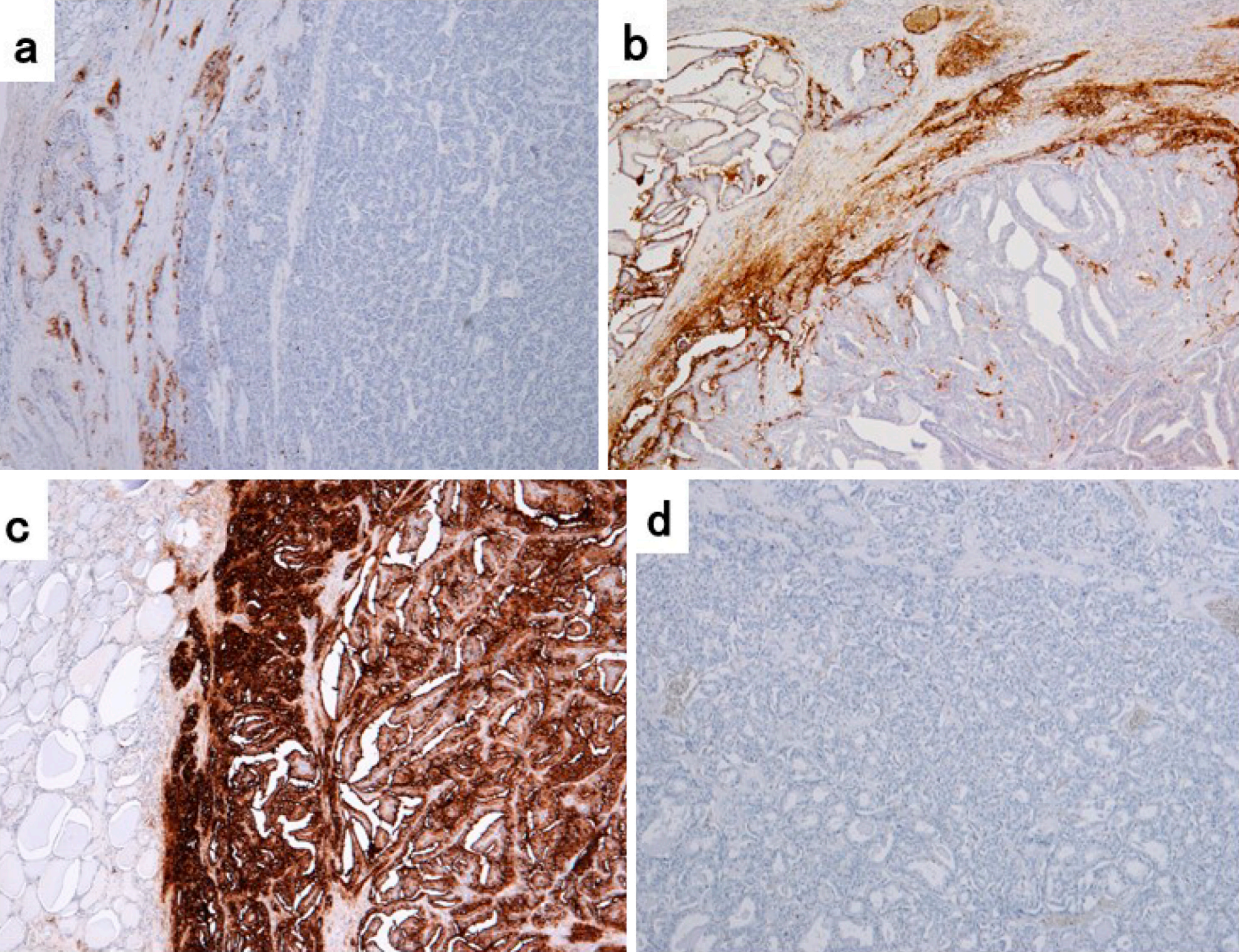
Immunohistochemistry for CA19-9 in the PTC tissues. Specimens with (a) weak, (b) moderate, (c) strong, and (d) almost no expression.

We performed CA19-9 immunohistochemistry on 62 archival formalin-fixed and paraffin-embedded specimens (12 from the High group and 50 from the Normal group). The immunohistochemical samples are shown in Figure 3. CA19-9 expression was detected in 28 of 62 PTCs (45.2%) at various degrees and ranges in the conventional PTC histology. The relationship between serum CA19-9 levels and CA19-9 immunostaining results is described in Table 2. Positive cytoplasmic staining was observed in 100% (12/12) of specimens in the High group, with 0% negative staining. In contrast, positive cytoplasmic staining was observed in only 32% (16/50) of the specimens in the Normal group. A statistically significant correlation was thus revealed between serum CA19-9 levels and the CA19-9 immunohistochemistry results (p < 0.01). Three patients whose tumor expressed CA19-9 had positive serum TgAb levels preoperatively (49.8, 58.0, and 72.3 IU/mL, respectively). Of 16 patients with positive immunohistochemical staining for CA19-9 and normal serum CA19-9 levels, two were serum TgAb-positive preoperatively (49.8 and 72.3 IU/mL, respectively). In the Normal group, there was no significant difference in the change in the serum CA19-9 levels before and after surgery in either positive (n = 16) or negative (n = 34) CA19-9 immunohistochemical staining results (the median serum CA19-9 level changed from 7.4 (range: 1.0-16.1) to 6.8 U/mL (range: 1.0-15.0), *p* = 0.40 or 10.9 (range: 1.0-34.6) to 11.0 U/mL (range: 1.0-29.5), *p* = 0.82).

**Table 2.** Relationship between Serum CA19-9 Levels and CA19-9 Immunostaining Results.

		Serum levels of CA19-9	
		High >37 U/mL	Normal ≤37 U/mL
CA19-9	Positive	12	16
Immunohistochemistry	Negative	0	34

### Follow-up and outcome

Only six of 28 patients with positive CA19-9 immunohistochemical staining results underwent radioactive iodine ablation (30mCi) within 1 year after surgery. At a median follow-up period of 55 months (range: 35-61 months), the mild serum Tg levels were elevated (0.8-2.6 ng/mL) in only four of 28 patients with the tumor expressing CA19-9. However, the structural recurrence based on imaging studies was not revealed. Serum Tg levels of the remaining patients were undetectable. In addition, the serum TgAb levels were undetectable in all patients.

## Discussion

Tg has been used for several decades as the primary biochemical tumor marker for patients with PTC ^(2), (3), (4), (5)^. Tg is a very sensitive and specific marker in patients who have undergone total thyroidectomy for PTC; disease progression correlates with increased serum Tg levels in patients with PTC. However, the distribution of Tg values is affected by the presence of TgAb, which interferes with Tg measurements, rendering the Tg level uninterpretable ^(6), (7), (8), (9), (10), (11)^. The prevalence of TgAb in patients with PTC is reported to be 25%, which is higher than that (10%) in the normal population ^(10), (11)^, and follow-up based on the Tg level is not appropriate for these patients. Therefore, no useful tumor markers are currently available to follow-up patients whos serum is TgAb-positive.

We treated a patient with multiple metastases of PTC and high serum CA19-9 levels, which is typically considered a gastrointestinal tumor marker ^(12)^. The patient’s serum CA19-9 and Tg levels, which were elevated preoperatively, were decreased after surgery, and the immunostaining examination for resected specimens revealed that the carcinoma cells of both primary and metastases showed positivity for both Tg and CA19-9. Thus, we speculated that serum CA19-9 levels might serve as a surrogate marker for PTC in place of the serum Tg level in patients for whom follow-up on the basis of Tg levels is not appropriate (such as patients with positive serum TgAb) since there is currently no useful tumor marker for use in patients whose serum is TgAb-positive.

There have been a few case reports on the relationship between PTC and serum CA19-9 as a tumor marker other than the case we reported ^(12), (15), (16)^. Nomizu et al. reported that serum CA19-9 showed low sensitivity as a tumor marker in a patient with PTC ^(16)^, but Yamaguchi et al. described a case in which serum CA19-9 was a useful tumor marker in a patient with PTC with lung metastases ^(15)^. Thus, it is not currently known whether CA19-9 is sufficiently reliable and useful as a tumor marker for PTC. Moreover, since serum levels of CA19-9 are not usually measured in patients with PTC, the percentage of elevated serum CA19-9 levels in patients with PTC is not known.

However, several research groups have described their detection of serum CA19-9 in patients with medullary thyroid carcinoma (MTC) ^(18), (19), (20)^. Elisei et al. ^(18)^ and Lorusso et al. ^(19)^ reported that an elevated serum CA19-9 level appears to be a predictive factor of poor prognosis in patients with advanced MTC and could be used to identify cases with a higher risk of mortality in the short term. Similarly, Alencar et al. reported that serum CA19-9 might have a role as a prognostic factor in patients with MTC ^(20)^. However, the precise relationship between PTC and serum CA19-9 levels has not been established. Thus, we conducted this study to determine the positive immunohistochemical staining rates for CA19-9 in PTC tissues and to clarify whether serum CA19-9 level is a useful tumor marker for PTC. To the best of our knowledge, this study is the first to retrospectively evaluate changes in serum CA19-9 levels in patients with PTC before and after thyroidectomy. Our present finding that 6% of the patients with PTC showed high serum CA19-9 levels before surgery and then normal serum CA19-9 levels after surgery is also a first report.

CA19-9 is an important and well-recognized tumor marker; elevated serum CA19-9 levels may indicate malignancies in the digestive tract, biliary tract, pancreas, or lungs. However, in our study patients, high serum CA19-9 levels decreased considerably and normalized after thyroidectomy, suggesting that the elevated CA19-9 levels before surgery were of thyroid origin rather than of pancreatic, gastrointestinal, or ovarian origin.

Although the serum CA19-9 levels of all patients in the Normal and High groups were within the normal range after surgery, serum Tg levels in all patients were decreased postoperatively compared with preoperatively, but some of the patients’ levels remained higher than normal. The difference in the half-lives of CA19-9 and Tg may explain this finding. The patients’ serum CA19-9 and Tg measurements were obtained before and at 0.5-1 month after surgery. The average half-life of CA19-9 is 0.5 days ^(21)^, whereas the half-life of serum Tg is several days, and its postoperative nadir was reported to be reached in almost all patients 3-4 weeks postsurgery ^(22), (23)^. After therapy with I-131, it takes several months for Tg to completely disappear from the circulation ^(24), (25)^. In fact, the study patients’ serum Tg levels were very low (0.1-1.1 ng/mL) or undetectable at 3 months postoperatively despite the lack of therapy with I-131.

We only enrolled the patients with a tumor diameter of >2 cm in the present analyses because serum CA19-9 and Tg levels may fail to identify patients with carcinoma lesions with a relatively small volume. There was no significant correlation between serum CA19-9 and Tg levels in the patients before and after surgery. We suspect that the reason for this lack of a correlation is that the degrees of CA19-9 expression differed among the patients. Inevitably, CA19-9 cannot be used as a tumor marker if its expression is weak or absent. It is important to note that in 12 of the patients, serum CA19-9levels, which were elevated before surgery, were decreased and normalized after surgery. This indicates that CA19-9 may be a tumor marker for PTC.

Using immunohistochemistry, Nishihara et al. confirmed the existence of the Tg protein in carcinoma lesions, even in patients with undetectable serum Tg levels ^(9)^. The details of the mechanism that underlies the clearance of Tg remain unclear; however, there are several reasons that a serum Tg level would be much lower than expected in a Tg assay, including dedifferentiation and somatic mutations in the *TG* gene in the tumor ^(9)^. In such cases, because Tg is less sensitive as a tumor marker, CA19-9 may be a surrogate tumor marker for PTC. However, if immunostaining for CA19-9 is negative in primary thyroid lesions and metastases, the patient’s serum CA19-9 level will not be an appropriate tumor marker. If Tg cannot be used as a tumor marker in patients with PTC (for example, if the patients’ serum is positive for TgAb or if serum TgAb is negative but the serum level of Tg is low), it would be worthwhile to perform immunostaining for CA19-9 in the patients’ carcinoma lesions to evaluate its potential as a surrogate tumor marker.

Changes in the quantity of the tissues with antigens should result in changes in the serum levels of antibodies ^(26)^. Postoperative changes in TgAb or specific TgAb levels can be a surrogate tumor marker for patients with PTC who are TgAb-positive after total thyroidectomy ^(17), (26), (27), (28)^. However, TgAb levels are influenced not only by the quantity, antigenicity, and viability of remnant carcinoma lesions, but also by residual thyroid tissue and presence of thyroid autoimmune disease ^(26)^. Therefore, TgAb levels are expected to vary among individuals, and its sensitivity and specificity as a tumor marker should be less than that of Tg ^(26)^. In contrast to Tg and TgAb, serum CA19-9 levels may be used as a surrogate tumor marker for patients with PTC regardless of the amount of residual thyroid tissue i.e., after hemithyroidectomy as well asand after total thyroidectomy.

This study has some limitations. First, CA19-9 measurement is not available for patients with thyroid cancer under the Japanese Health Care Insurance System and its cost is extremely high. Thus, we could only measure CA19-9 once each, before and 0.5-1 month after surgery. Second, because our patient population excludes any cases of recurrence as of this writing, we have not been able to confirm whether the serum CA19-9 levels were elevated again after recurrence. Third CA19-9 is a sialylated Lewis A (sLea) structure in which sialic acid is attached to the Lewis A carbohydrate chain (Lea) of a Lewis blood group, and a fucosyl is attached to the CA19-9 precursor (sialyl LeC). The reaction requires α1-4 fucosyl transferase, but this enzyme is deleted in the Lewis blood group (a−b−); thus, sLea (CA19-9) cannot be produced, and serum CA19-9 is low. Serum CA19-9 levels therefore differ depending on the the Lewis blood group phenotype ^(29)^, but the Lewis antigen was not evaluated in the present study. Further studies are necessary with longer follow-ups to expand our knowledge about the significance of CA19-9 in patients with PTC.

In conclusion, to the best of our knowledge, this is the first study to retrospectively evaluate the percentage of CA19-9 positivity in PTC tissues and the pre- and post-thyroidectomy changes in serum CA19-9 levels in patients with PTC. Notably, 6.1% of the patients showed serum high levels of CA19-9 before surgery. Although further studies with longer follow-ups are necessary, positive immunostaining for CA19-9 in carcinoma lesions indicates that serum CA19-9 levels may serve as a surrogate tumor marker for PTC in place of the serum Tg levels in some patients if Tg cannot be used as a tumor marker as in patients who are TgAb-positive.

## Article Information

### Conflicts of Interest

None

### Author Contributions

MK and AM contributed to the conception and design of the study. MK, MH, and AS performed data acquisition. MK performed data interpretation and drafted the manuscript. AM, MH, AS, and TA critically revised the manuscript. All authors declare that they contributed to this article and have read and approved the final version.

### Approval by Institutional Review Board (IRB)

This study was approved by our hospital’s institutional ethics committee (no. 20180517-1).
